# Peripheral Nerve Stimulation for Treatment of Cluneal Neuropathy Case Study

**DOI:** 10.7759/cureus.28033

**Published:** 2022-08-15

**Authors:** Costa Soteropoulos, Joseph Pergolizzi, Sindhu Nagarakanti, Christopher Gharibo

**Affiliations:** 1 Pain Management, National Spine & Pain Centers, Fredericksburg, USA; 2 Cardiology, Native Cardio, Naples, USA; 3 Neuroscience, Temple University, Philadelphia, USA; 4 Pain Management, New York University (NYU) Langone Health, New York City, USA

**Keywords:** interventional pain medicine, lower back pain, axial spine, acute pain, cluneal neuralgia

## Abstract

Chronic low back pain is a prevalent and sometimes debilitating condition. This case report describes a 69-year-old female presenting with axial spine pain. The pain was inadequately controlled by opioids as she was treated unsuccessfully with hydrocodone and remained to have the pain between 7/10 and 10/10. Peripheral neural stimulation (PNS) was trialed and then used to control her pain. PNS is a device-based treatment option that appears effective in a subset of patients. It has been effectively used to treat many different chronic pain syndromes. The patient responded well to the treatment, with her pain intensity going down to between 2/10 and 5/10 on the same scale. She was able to discontinue her use of opioids. PNS can be a safe and effective treatment in patients who have not responded well to pharmacologic analgesia.

## Introduction

Peripheral nerve stimulation is a commonly used process that helps treat chronic lower back pain. It commonly involves surgery, in which the physician places a small electrical device next to one of the peripheral nerves. This device sends small electric pulses that aid in the management of the pain. 

Some conditions for which PNS are used are median/ulnar/radial neuropathy, occipital neuralgia, cluneal nerve pain, pudendal neuralgia, femoral/sciatic/obturator neuropathy, brachial/lumbar plexus neuropathy, meralgia paresthetica, lumbar/cervical radiculitis, and intercostal neuralgia. There have been tremendous advancements in PNS, such as improvements in ultrasound-guided imaging, integration of ultrasound into clinical practice, percutaneous implantation techniques, smaller devices, and rechargeable and larger-capacity batteries. Unfortunately, there is very little evidence proving the long-term effects of PNS with regard to managing chronic pain. PNS offers a promising and nonpharmacologic treatment to chronic pain that in some cases may reduce or even eliminate the need for pharmacologic therapy [1].

Lower back pain is aching or shooting pain that affects the area of the back that starts below the ribcage. Causes of this pain include muscle strain, spinal stenosis, which is the narrowing of the space surrounding the spinal cord, ankylosing spondylitis, the inflammation of the spine joints, and fibromyalgia, which causes widespread muscle pain, including the pain of the lower back. Risk factors for lower back pain include obesity, being sedentary, and heavy lifting. Prevention includes healthy eating and regular exercise [1].

Cluneal nerve neuropathy is a known cause of lower back pain. It is the entrapment of the middle cluneal nerves which induces symptoms of pain in the lower back and legs. When the cluneal nerve passes under the long posterior sacroiliac ligament, the nerves spontaneously become entrapped. It is an underdiagnosed cause of lower back pain, and because of this, unnecessary spinal surgeries and sacroiliac fusions are conducted. Not much literature contains a plethora of information on cluneal nerve neuropathy [2].

## Case presentation

A 69-year-old female presented with axial spine pain secondary to bilateral sacroiliitis, bilateral lumbar facet joint arthropathy, and right lower back pain medial to the sacroiliac joint and inferior to the facet joint line for over 10 years. She reported pain of anywhere from 7/10 to 10/10 on a VAS scale. The pain was inhibiting her daily life as it affected her mobility and transfers, leading her to use a cane. 

Her pain was managed using a multimodal regimen which included physical therapy, NSAIDs, gabapentin, hydrocodone, chlorzoxazone, tramadol, and various opioids, with minimal pain relief. The patient took one 10 mg hydrocodone tablet thrice a day, as recommended, before the procedure to help with the pain. Later she underwent medial branch blocks and radiofrequency ablation in her lumbar spine, sacroiliac joint injections, epidurals, and sacroiliac radiofrequency ablation with some good results; however, she continued to have persistent non-specific residual right lower back pain associated with the medial cluneal nerves.

Despite all interventions, she continued to experience chronic low back pain. Further clinical evaluations revealed cluneal neuropathy. This condition was treated with a right-sided peripheral nerve stimulator (PNS) trial medial to the right sacroiliac joint, which resulted in nearly 80% elimination of her residual pain. After that, she was implanted with the PNS stimwave device in the same place, which continued to give her 80% or more durable relief in her residual right lower back pain. The lead was inserted 4 to 5 cm medial to the sacroiliac joint over the dorsal branches sacral (S)1, S2, S3, and S4. 

The patient had marked pain reduction and was able to discontinue the hydrocodone. Her function and quality of life have improved. When her pain was reassessed, she reported pain intensity of 2/10 to 5/10 on the same scale.

## Discussion

Over one-quarter of Americans suffer from chronic pain, which can result in significant mortality. Any one treatment for this pandemic has many shortfalls making it so that no one treatment can cure all of any patient's problems. Opioids are effective chronic pain relievers but are associated with a constellation of opioid-related side effects that can limit treatment. Among these is opioid use disorder (OUD) which affects over 16 million people. In addition, opioid prescribing has been associated with opioid misuse and abuse. According to the Centers for Disease Control (CDC), 39 people die every day of a drug overdose involving an opioid. As a result, finding safe, effective ways to treat severe chronic pain is crucial. Multimodal therapy is a combination of two or more modalities that target different parts of the disease. Both must be regulated, as although they might only have modest effects when used alone, together, their effects can be more effective [3]. Interventional pain techniques under imaging guidance are an example of a multimodal therapy that has promising results. Some examples include epidural steroid injections, nerve blocks, radiofrequency ablation, spinal cord stimulation, facet joint injections, lumbar sympathetic plexus blocks, and trigger point injections. PNS, another example, is a device-based treatment that has been shown effective in treating low back pain and many other painful conditions [4]. Lower back pain (LBP) is one of the chief sources of chronic pain and globally, according to the World Health Organization (WHO), is the number one cause of disability. It affects around 8% of all adults globally, which is around 577 million adults [1]. It has been associated with healthcare-related expenditures of around $380 billion and costs in loss of productivity [5]. Not all patients, though, respond to PNS and may have to be trialed before the implant to see if they’re responders. The nerves are located within the body, so the procedure is considered minimally invasive.

As it involved a minimally invasive procedure to place the device, there is also a risk of device-related complications. The success rate is around 80%, with most complications arising from more complicated issues rather than cluneal nerve neuropathy [6].

## Conclusions

LBP is a debilitating chronic condition that affects a large subpopulation. This case study found that PNS was a safe, effective treatment for axial lower back pain secondary to cluneal neuropathy to the bilateral sacroiliitis, bilateral lumbar facet joint arthropathy, and right lower back pain medial to the sacroiliac joint and inferior to the facet joint line. Although more studies are needed, the patient was satisfied with the pain control provided by PNS and was able to discontinue her hydrocodone pharmacotherapy. 
